# Deep learning-based 2D/3D registration of an atlas to biplanar X-ray images

**DOI:** 10.1007/s11548-022-02586-3

**Published:** 2022-03-16

**Authors:** Jeroen Van Houtte, Emmanuel Audenaert, Guoyan Zheng, Jan Sijbers

**Affiliations:** 1 imec-Visionlab, University of Antwerp, 2610 Antwerp, Belgium; 2 Department Human Structure and Repair, University Ghent, 9000 Ghent, Belgium; 3 Department of Electromechanics, Op3Mech Research Group, University of Antwerp, 2020 Antwerp, Belgium; 4 Institute of Medical Robotics, School of Biomedical Engineering, Shanghai Jiao Tong University, Shanghai, 200240 China; 5 µNEURO Research Centre of Excellence, University of Antwerp, 2610 Antwerp, Belgium

**Keywords:** Deep learning, Digitally reconstructed radiographs, X-ray imaging, 2D/3D image registration, Image warping, Atlas image

## Abstract

**Purpose:**

The registration of a 3D atlas image to 2D radiographs enables 3D pre-operative planning without the need to acquire costly and high-dose CT-scans. Recently, many deep-learning-based 2D/3D registration methods have been proposed which tackle the problem as a reconstruction by regressing the 3D image immediately from the radiographs, rather than registering an atlas image. Consequently, they are less constrained against unfeasible reconstructions and have no possibility to warp auxiliary data. Finally, they are, by construction, limited to orthogonal projections.

**Methods:**

We propose a novel end-to-end trainable 2D/3D registration network that regresses a dense deformation field that warps an atlas image such that the forward projection of the warped atlas matches the input 2D radiographs. We effectively take the projection matrix into account in the regression problem by integrating a projective and inverse projective spatial transform layer into the network.

**Results:**

Comprehensive experiments conducted on simulated DRRs from patient CT images demonstrate the efficacy of the network. Our network yields an average Dice score of 0.94 and an average symmetric surface distance of 0.84 mm on our test dataset. It has experimentally been determined that projection geometries with 80$^{\circ }$ to 100$^{\circ }$ projection angle difference result in the highest accuracy.

**Conclusion:**

Our network is able to accurately reconstruct patient-specific CT-images from a pair of near-orthogonal calibrated radiographs by regressing a deformation field that warps an atlas image or any other auxiliary data. Our method is not constrained to orthogonal projections, increasing its applicability in medical practices. It remains a future task to extend the network for uncalibrated radiographs.

## Introduction

Radiography or X-ray imaging is the most common imaging procedure for many orthopaedic interventions thanks to its ability to visualise internal structures with a relatively low radiation dose and low acquisition cost. Apart from diagnosis, it is a valuable imaging technique for intraoperative guidance and post-operative evaluation. It also plays a crucial role in pre-operative surgical planning and the selection of the right implants. In case of total hip arthroplasty surgeries, for example, it has been shown that the proper positioning and orientation of the acetabular component largely determines the functional outcome of the implant [1, 2]. Thereby, it is essential that parameters such as the centre of rotation of the hip joint, leg length, and hip offset remain preserved after the surgery and thus are correctly assessed on the radiographs.

Although many surgical planning tools rely on two-dimensional (2D) radiographs, their clinical interpretation can be hampered by overlapping structures and magnification effects. The assessment from radiographs can also be influenced by the patient’s positioning. To avoid the difficulties associated with 2D projections, three-dimensional (3D) computed tomography (CT) images are preferred for surgical planning because they are less ambiguous [3]. They also allow to study the cortical and cancellous bone, in addition to the outer bone surface [4]. CT-based planning, however, is associated with higher radiation doses and to far more expensive image acquisitions. Previous research has therefore suggested the reconstruction of a patient-specific 3D model from two or more 2D radiographs by registering a 3D CT atlas image to 2D radiographs, referred to as 2D/3D registration [4].

## Related work

Recently, deep-learning (DL) methods have been proposed that reconstruct a 3D image from 2D radiographs by means of a neural network that encodes the 2D radiographs into a latent variable which is decoded into a 3D CT volume [5–8]. Compared to 3D/3D registrations, these networks need to bridge between the different dimensionalities in the encoder and decoder, which can be done by reshaping the 2D feature maps [5] or by treating the feature channel as the third spatial dimension [6]. Others exploit the orthogonality between biplanar projections by copying each feature map along a different dimension [7]. The X2CT-GAN network uses two different mechanisms to bridge the dimensionalities [8]. They apply a fully connected layer on the flattened latent variable, before applying a nonlinear activation function and reshaping it into a 3D feature map. For the skip connections, they apply 2D convolutions on the 2D feature maps, which are then copied along the third axis and fed into a 3D convolutional layer.

In this paper, we propose a novel atlas-based 2D/3D registration network that estimates a registration field based on a pair of calibrated radiographs. The main contributions of our proposed method are as follows:

- It follows a registration approach instead of a reconstruction approach, by regressing a deformation field which can be used to warp an atlas or any auxiliary data like segmentation maps. This avoids an additional segmentation step to extract a surface model.
- It decomposes the total registration function into an affine and a local part in order to reduce restrictions on the orientation of input data.
- It is not restricted to orthogonal projections, unlike other DL-methods in the literature. To this end, we propose an inv-ProST layer to better combine bi-directional feature maps, as an extension to [9].
- It is validated on simulated digitally reconstructed radiographs (DRRs) from a large collection of patient CT images, and compared to other registration approaches in the literature [7, 10].

## Methodology

### Registration network architecture

**Fig. 1:**
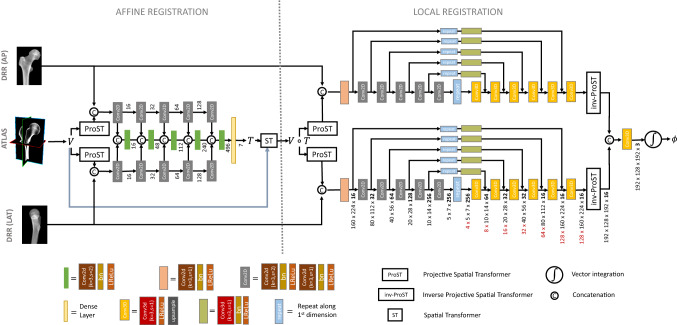
Architecture of the 2D/3D registration network consists of an affine and local registration module. The affine module regresses the 7 affine parameters of the transformation *T* by encoding the anterior-posterior (AP) and lateral (LAT) radiographs. The atlas image *V* is warped by the regressed transformation before being fed into the local registration module, which regresses the 3D local deformation field $\phi$ by encoding and decoding the AP and lateral radiographs separately

#### Overview of network

The registration network, shown in Fig. 1, estimates a registration field $\Psi$ that maps the atlas image *V* (with associated label map *S*) to the moving image space, such that the forward projection of the warped atlas, $V\circ \Psi$, matches the input radiographs $I_i$, with $i \in \{\text {AP}, \text {LAT}\}$. The registration field $\Psi$ can be decomposed into an affine transformation *T* and a local backwards deformation field $\phi$. Both transformations are separately regressed by two sequential network modules and composed at the end of the network to yield the total deformation field $\Psi = \phi + T \circ \phi$, which is used to warp the atlas image.

#### Projective spatial transform layer

The projective spatial transformer (ProST), introduced by Gao et al. [9], simulates a 2D perspective projection ${\hat{I}} \in \mathrm{I\!R}^{S_x\times S_y }$ from a 3D volume *V* by sampling this volume at grid locations $G \in \mathrm{I\!R}^{S_x\times S_y \times K }$. The grid consists of *K* sampling points, uniformly distributed along each ray connecting the X-ray source location to each pixel of the 2D detector. This canonical grid can be transformed by an affine transformation $T_{geom}$ in order to represent the actual projection geometry. This projection geometry transformation $T_{geom}$ is known for calibrated radiographs and serves as input parameter to the network. The 3D volume *V* can be interpolated at the transformed grid positions $T_{geom}(G)$ to obtain an X-ray beam-aligned image volume $V_{beam} \in \mathrm{I\!R}^{S_x\times S_y \times K }$ in the beam-space:

1 $$\begin{aligned} V_{beam} = V \circ (T_{geom}(G)). \end{aligned}$$

The cone-beam projection is then obtained by integration along each ray, which is equivalent to a “parallel projection” of the interpolated volume:

2 $$\begin{aligned} {\hat{I}}^{(i,j)} = \sum _{k=1}^K V_{beam}^{(i,j,k)}. \end{aligned}$$

#### Affine registration module

The affine registration network consists of two ProST layers which project the 3D atlas image along the AP and lateral direction. The ProST output ${\hat{I}}_i$ and the input radiograph $I_i$ are concatenated into a 2-channel 2D image and fed into a 2D encoder, corresponding to the *i*th projection direction. Each of the five encoder levels consists of a strided convolution, a batch-normalisation layer and a leaky rectified linear activation unit (Leaky-ReLU). Each level reduces the spatial size of the feature map by a factor two and doubles the number of features. At each encoder level, the AP and lateral features (and the preceding combined features) are concatenated and convolved.

The accumulated 2D feature map at the last encoder level is flattened and fed into a dense layer which regresses the seven parameters of the affine transformation *T* between the floating image and the atlas. The bias and kernel weights of the dense layer are initialised by zero and a narrow normal distribution, respectively, such that the initial affine transformation during training is close to identity. A spatial transform layer warps the atlas image *V* by the affine transformation *T* [11], before being fed to the local registration network.

#### Local registration module

The local registration network consists of two separated U-net-shaped networks, each associated with a different projection direction. Each U-net-shaped network is composed of a 2D encoder and 3D decoder and is preceded by a ProST layer that projects the affine transformed atlas image. Each level of the 2D encoder consists of a strided and non-strided 2D convolution. By consequence, each level halves the spatial size and doubles the number of features of the feature maps. After each 2D convolution, a batch normalisation and a Leaky-ReLU activation are applied. The last 2D feature map is copied $M=4$ times along the first dimension to obtain a 3D feature map.

The spatial dimensions of the 3D feature map are increased by the 3D decoder, while reducing the number of features as follows: [64, 32, 32, 16, 16, 16]. Each decoding step applies a 3D convolution with stride one and a Leaky-ReLU activation, followed by upsampling the feature map by a factor of two. The 3D feature maps are defined in the beam-space, which gives a natural meaning to the above operations. While stacking 2D maps corresponds to increasing the number of sampling points per ray, the upsampling also increases the number of rays.

The network has skip connections between the 2D encoder and 3D decoder at each resolution level of the U-shaped network in order to recover spatial information loss that might have happened during down-sampling. Along each skip connection, the 2D feature maps are copied along the first dimension a number of times, such that its shape corresponds to the 3D decoded feature map’s shape. After copying the feature map, the skip connection applies a 3D convolution, a batch normalisation, and a Leaky-ReLU activation to the feature map.

#### inv-ProST

The decoded 3D feature maps are defined in the beam-space and need to be converted to physical space to align them with each other before combining them. Therefore, we apply an “inv-ProST” layer to the 3D feature maps, which samples the feature maps at locations $G^{-1}$:

3 $$\begin{aligned} {\hat{V}} = V_{beam} \circ (T_{geom}(G^{-1})), \end{aligned}$$

with $G^{-1}$ the canonical sampling coordinates in the beam space, which are determined by the length of the rays connecting the source location with each voxel and by the intersection point of those rays with the detector plane.

It can be verified that successively applying the ProST of Eq. (1) and inv-ProST of Eq. (3) on an image volume *V* results in approximately the same image *V* apart from interpolation approximations. Only voxels in the original image that fall outside the cone-beam become zero in the final image.

After the inv-ProST layer, the output tensors of the AP and the lateral network branches can be combined by concatenation, and convolved into a 3-channel tensor which is interpreted as a stationary velocity field. This velocity field is integrated by a “scaling and squaring”-method to obtain a diffeomorphic deformation field $\phi$ [11, 12].

### Semi-supervised learning

The training of the network is semi-supervised, which means that the training of the network relies on auxiliary data. In our experiment, the segmentation labels of the ground-truth CT volumes were used to mask the image volumes before feeding them into the network, as we are only interested in reconstructing the femur bone from the radiograph images.

The registration quality of the end-to-end network during training and validation is quantified by a loss function that consists of a normalised cross-correlation (NCC) function between the ground-truth image volume $V^f$ and the warped atlas image *V* after the affine and local registration. Furthermore, it contains a regularisation term on the smoothness of the local deformation field:

4 $$\begin{aligned} {\mathcal {L}}&= - {\mathcal {L}}_{NCC}(V \circ T, V^f) - {\mathcal {L}}_{NCC}(V \circ \Psi , V^f) \nonumber \\&\quad + \delta {\mathcal {L}}_{smooth}(\phi ) \end{aligned}$$

The hyper-parameter $\delta =0.01$ balances the contribution between the smoothness term and the image similarity loss. Note that the loss function does not include the label maps anymore, as the images themselves are already masked.

## Experiments

In this section, we evaluate the performance of our network. “Experimental settings” section discusses the generation of the different datasets and provides details on the evaluation and training procedure. “Experimental results” section presents the registration results for AP and lateral radiographs while comparing with other methods. We also report the sensitivity to inaccurate input parameters and the accuracy for non-orthogonal projections. An ablation study is presented in “Ablation study”.

### Experimental settings

#### CT-data preprocessing and augmentation

A total of 315 angio-CT images were acquired and split into a training set of 235 subjects, a validation set of 40 subjects for model selection, and a test set of 40 subjects used to report performance. From each CT-image, the left and right femurs were extracted and rotated to a reference system that aligns the anterior-posterior and lateral views of that femur with the *x* and *y*-axis of the image. The femur reference frame of each image was defined based on the neck and shaft axis of the femur. To allow some pose variation around this canonical reference pose, we applied random affine transformations to the image with strict constraints. The randomised angles were allowed within a range of 10$^{\circ }$ extension/flexion, 10$^{\circ }$ abduction/adduction and 10$^{\circ }$ internal/external rotation.

After transforming the images to a pose that is close to that of the reference, the images were cropped around the femoral heads and resized in order to maintain the highest resolution as possible. The left femur images were flipped to resemble right ones. The final CT volumes have a size equal to $(192 \times 128 \times 192)$, and a resolution of $(0.664 \times 0.664 \times 1)$ mm$^3$. Each image has a corresponding segmentation map *S*, obtained by graph-cut segmentation method followed by manual corrections [13].

#### Generating DRR

Digitally reconstructed radiographs (DRR) were simulated from the femur-centred CT volumes by DeepDRR software [14]. DRRs were created with an image size of (422 $\times$ 640), and downsampled to (160 $\times$ 224) to fit the network’s input size. The source-detector distance and the isocentre distance of the projection geometry were fixed to 1000 mm and 925 mm, respectively. Two different datasets of DRRs were generated:

- A dataset with orthogonal projections. The projection geometry was fixed to provide lateral and AP projections. The acquisition geometry corresponding to this dataset resembles best the experimental settings in the literature.
- A dataset with generalised projection geometries. Projection matrices were parameterised by the left/right anterior oblique (LAO/RAO) angle $\theta$, which was randomly varied between − 30$^{\circ }$ and $+$ 30$^{\circ }$, around the perfect lateral and AP view. The cranio-caudal angle was set to a constant value of 0$^{\circ }$. Different combinations of LAO/RAO angles were made for biplanar experiments.

For both datasets, the CT label maps were projected along with the CT images to obtain a 2D labelmap for the DRRs. The DRRs were masked by these labelmaps before feeding them into the network. Note that other structures, in front and behind the femur, are still visible in the masked DRRs.

#### Evaluation metrics

The registration accuracy of the network is evaluated by means of the Dice score and the Jacard coefficient, which measure the overlap between the warped atlas label map and the ground-truth label map [15]:

5 $$\begin{aligned} Dice(A,B)&= 2\frac{\vert {A\cap B}\vert }{\vert {A}\vert +\vert {B}\vert } \end{aligned}$$

6 $$\begin{aligned} Jac(A,B)&= \frac{\vert {A\cap B}\vert }{\vert {A \cup B}\vert } \end{aligned}$$

We also report the average symmetric surface distance (ASSD), which measures the average geometric distance between the ground-truth and registered bone surfaces. The similarity between the warped atlas image and the ground-truth image volume is quantified by the structural similarity index (SSIM), which takes the luminance, contrast and structure into account. As our method is a registration method, its ability to estimate the right intensity values of the image volume is limited. It can only warp an atlas with fixed intensity values.

#### Training details

We implemented our network by using the TensorFlow library. The network was trained for 300 epochs on a NVIDIA Tesla A100 graphics card. The model requires 18.7 GB of memory when being trained with a batch size equal to one, and has a computational complexity of 722 GFLOPS. The loss-function was minimised using the Adam optimizer, with the learning rate set to $10^{-5}$.

**Fig. 2:**
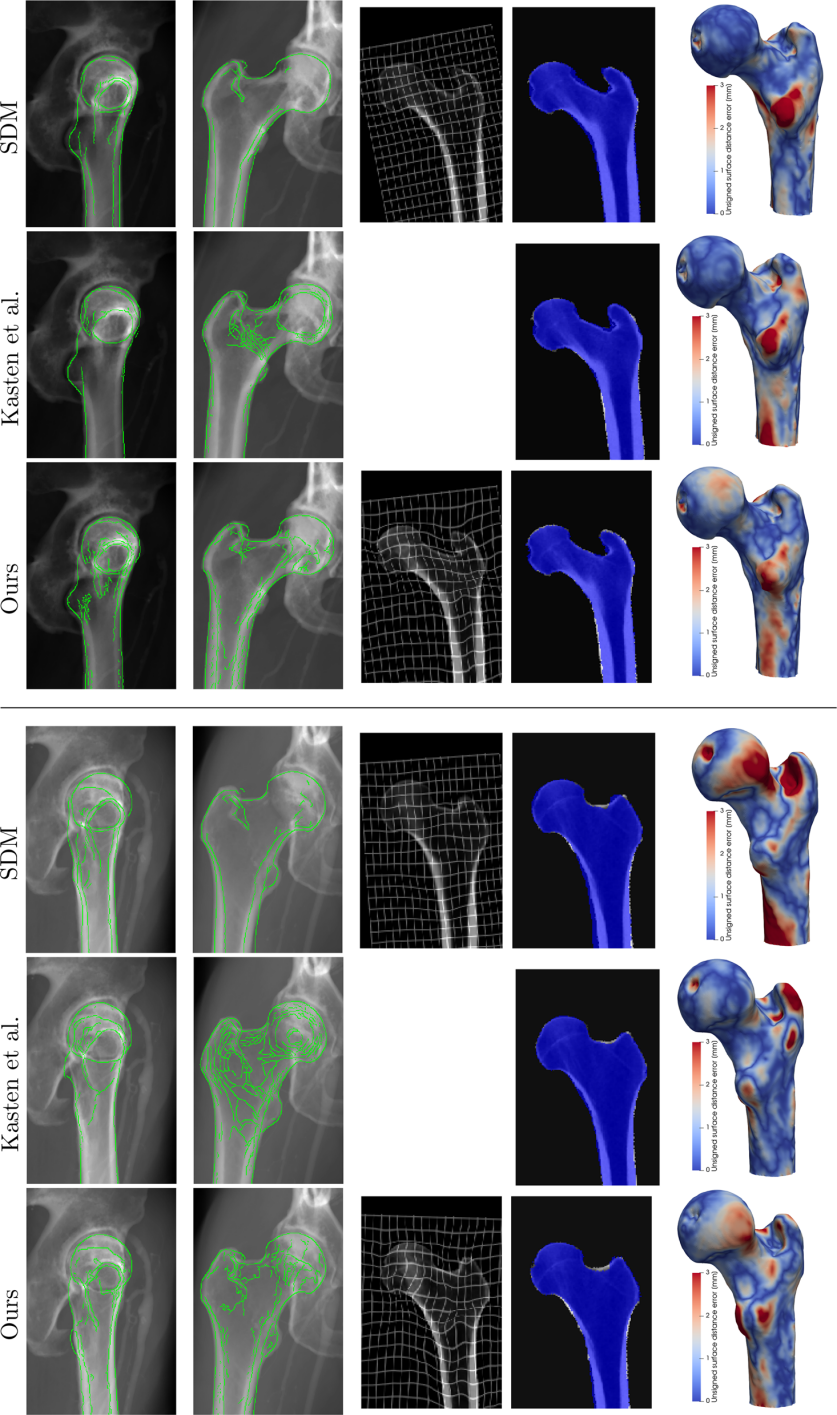
Examples of 2D/3D registration based on AP and lateral radiographs by different DL models. The first two columns show the lateral and AP input radiographs overlapped with the contours of the DRRs from the predicted 3D image volume. The third column shows a coronal slice of the warped atlas volume with the deformation grid. The fourth column shows a coronal slice of the predicted segmentation map, overlaid on top of the ground-truth image. The last column shows the geometric reconstruction error between the reconstructed and ground-truth surface model

### Experimental results

#### Comparison with other methods

This section describes the results of the registration to AP and lateral DRRs, by our proposed network and by two other networks for comparison. The evaluation metrics are listed in Table 1. Figure 2 illustrates the qualitative performance of the network by some registration examples.

The first comparison method registers a B-spline-based statistical deformation model (SDM) to a pair of radiographs by regressing its principal component weights [10]. This is a deep-learning implementation of the classical method of Yu et al.(2017) [4]. The SDM guarantees plausible shapes and provides smoother deformation fields than our proposed method, as can be seen in Fig. 2. Nevertheless, it is outperformed by our method in terms of registration accuracy ($p=10^{-30}$), as reported in Table 1. This indicates that the constraint on the deformation field by the SDM is too strong to correct for small-scale deformations. The lower SSIM value is due to the different atlas image being used for the SDM-based method. This atlas has an average intensity profile which cancels out more subtle local intensity variations.

The second comparison method is a re-implementation of the work of Kasten et al. [7], in which the 3D binary labelmap of the femur is immediately regressed from the biplanar radiographs, without deforming an atlas image. This method achieves a larger Dice score than our method ($p=4\cdot 10^{-4}$), but lacks information about the internal structures. As it does not regress the 3D intensity values, the problem is considerably simplified.

**Table 1:** Registration accuracy of our proposed method and comparison methods [7, 10]

	Dice	Jac	SSIM	ASSD
SDM [10]	0.921 ± 0.017	0.854 ± 0.028	0.327 ± 0.083	1.16 ± 0.21
Kasten et al. [7]	0.943 ± 0.015	0.892 ± 0.025	–	0.83 ± 0.18
Ours	0.939 ± 0.016	0.886 ± 0.027	0.932 ± 0.013	0.84 ± 0.20

Figure 2 shows a good alignment for our method between the input DRRs and the simulated perspective projections of the registered atlas images, including the cortical bone. The geometric distance error between the estimated and ground-truth surface model highlights the lesser trochanter as a challenging region to register accurately for all methods, while global structures like the femoral neck and shaft are more accurately reconstructed.

**Fig. 3:**
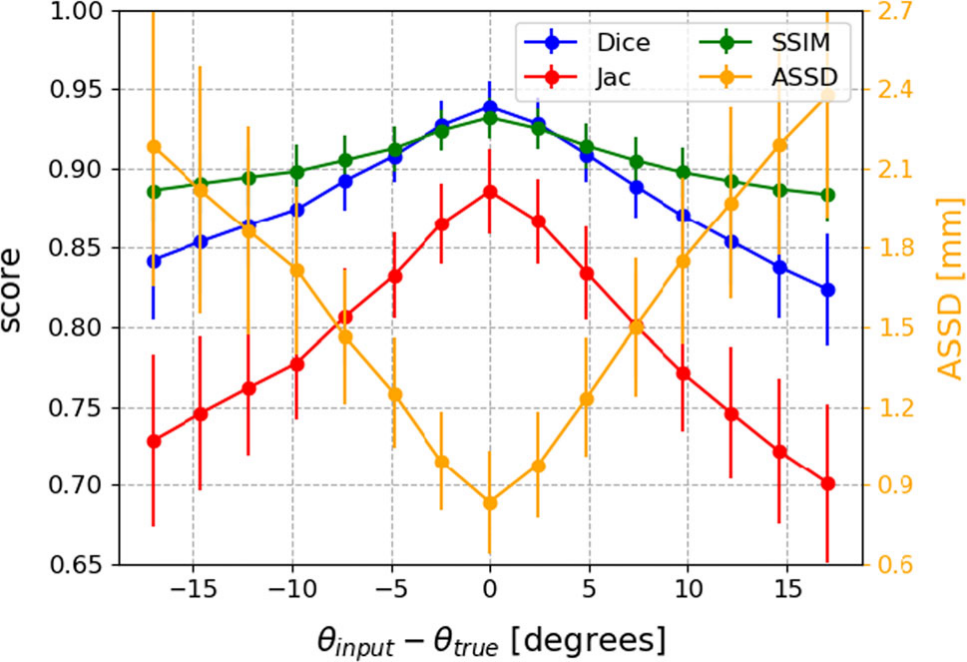
Sensitivity of the registration accuracy to inaccurate LAO/RAO projection angle inputs. The true angles $\theta _{true}$ in this experiment correspond to perfect AP and lateral views

#### Sensitivity to inaccurate input

Our network requires calibrated radiographs as input, meaning that the corresponding projection matrix, parameterised by the intrinsic and extrinsic parameters, needs to be known. However, the orientation of an imaging system, like a C-arm system, can never exactly be determined in practice, especially if both projections are taken at different times and the patient moves in between both acquisitions. In this experiment, we study how the uncertainty on the LAO/RAO projection angle affects the registration accuracy for projections which are in reality orthogonal. Figure 3 shows the evaluation metrics with respect to the difference between the ground-truth and input projection angle. For a discrepancy of 5$^{\circ }$, the average dice score gets reduced from 0.94 to 0.90.

#### Generalised projection geometries

We retrained and evaluated the registration network on the DRR dataset with generalised projection angles. Instead of perfect AP and lateral DRRs, projections were randomly generated in a range of 60$^{\circ }$ around the AP and lateral views. By training the network on such generalised dataset, the network can be reused for any projection geometry.

**Fig. 4:**
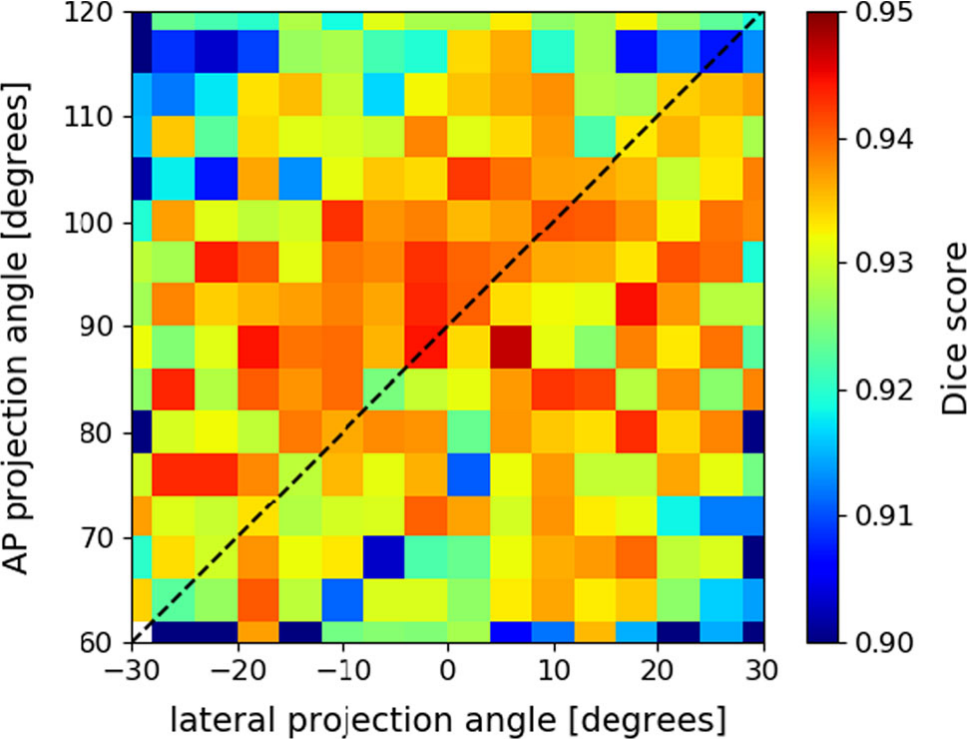
Dice scores for different biplanar configurations. Projection angles vary 60$^{\circ }$ around the perfect AP and lateral angle. The dashed diagonal line shows the configurations with 90$^{\circ }$ difference between the two projection directions. The bin size is 4$^{\circ }$

The overall average dice score on the generalised validation dataset ($N=2880$) equals $0.923\pm 0.033$. Figure 4 shows the median Dice scores for different combinations of LAO/RAO projection angles. The Dice score is maximal for near-orthogonal projection geometries, where the angle between both projection directions is between 80$^{\circ }$ and 110$^{\circ }$. It is interesting to note that projections do not necessarily need to correspond to perfect AP and lateral views.

### Ablation study

To study the effectiveness of individual components in our registration network, we re-trained our network, omitting some modules. We used the same dataset as in “Experimental results” section for training, validation, and testing. The evaluation metrics, listed in Table 2, are compared to the original results of “Experimental results” section by means of a two-sided paired t-test.

**Table 2:** Quantitative results for the effectiveness of different network components

	Aff				Aff+local			
	Dice	Jac	SSIM	ASSD	Dice	Jac	SSIM	ASSD
Original	0.860	0.755	0.893	2.04	0.939	0.886	0.932	0.84
	(0.024)	(0.036)	(0.015)	(0.35)	(0.016)	(0.027)	(0.013)	(0.20)
2 aff encoders	0.855	0.748	0.891	2.13	0.940	0.888	0.933	0.82
	(0.023)	(0.035)	(0.016)	(0.34)	(0.016)	(0.027)	(0.013)	(0.20)
Wo skip	0.846	0.734	0.887	2.29	0.937	0.883	0.931	0.86
	(0.035)	(0.050)	(0.016)	(0.52)	(0.017)	(0.029)	(0.013)	(0.21)
Single 3D dec	0.853	0.744	0.890	2.16	0.930	0.870	0.925	0.97
	(0.021)	(0.032)	(0.017)	(0.33)	(0.018)	(0.030)	(0.014)	(0.24)
Wo inv-ProST	0.851	0.742	0.889	2.18	0.932	0.873	0.927	0.95
	(0.026)	(0.038)	(0.015)	(0.36)	(0.016)	(0.027)	(0.013)	(0.21)
2 aff encoders	$10^{-7}$	$10^{-7}$	$10^{-7}$	$10^{-9}$	$10^{-2}$	$10^{-2}$	$10^{-1}$	$10^{-2}$
Wo skip	$10^{-8}$	$10^{-8}$	$10^{-9}$	$10^{-7}$	$10^{-3}$	$10^{-3}$	$10^{-3}$	$10^{-3}$
Single 3D dec	$10^{-10}$	$10^{-10}$	$10^{-8}$	$10^{-11}$	$10^{-21}$	$10^{-21}$	$10^{-23}$	$10^{-22}$
Wo inv-ProST	$10^{-12}$	$10^{-13}$	$10^{-13}$	$10^{-12}$	$10^{-17}$	$10^{-17}$	$10^{-16}$	$10^{-17}$

The mean and standard deviation (between brackets) of the evaluation metrics are tabulated for the different network variations. The bottom table shows the p-values of a paired t-test between the original network and each variation on the network architecture

#### Effectiveness of affine network structure

In this experiment, the affine network of “Affine registration module” section was modified by removing the intermediate concatenations of AP and lateral feature maps. Instead, they were only combined at the end of the affine module, right before regressing the affine parameters. While the affine initialisation is significantly worsened by this, the local registration remains unaffected. It shows that the local registration has a large enough capture range to correct for variations left unseen by the affine initialisation.

#### Effectiveness of skip-connections

Removing the skip connections in the local network significantly reduces the registration accuracy $(p=10^{-3})$. Secondly, it also increases the training time from 300 to 700 epochs, especially due to the slower training of the affine network. The mismatch in learning rate between the affine and local network can be explained by the vanishing gradient problem. In deep neural networks, the gradient might become very small for the early layers in the network, resulting in a negligible parameter update. The skip connections provide an alternative path to back-propagate the loss-function, which is essential for updating the early network layers.

#### Effectiveness of two separate 3D decoders

Instead of treating the AP and lateral feature maps separately by two distinct encoder-decoder modules, this network variation combines both feature maps at each level of the 2D encoder, similar to the affine network structure, and only contains one 3D decoder. Skip connections are included between the combined 2D feature maps and 3D decoder. The affine registration module remains the same as depicted in Fig. 1. The results in Table 2 show a highly significant reduction in the affine and local registration accuracy, indicating the preference to decode the 3D feature maps for each projection direction separately.

#### Effectiveness of inv-ProST layer

The inv-ProST layer is responsible for spatially aligning the decoded 3D feature maps into a common coordinate system, before regressing the deformation field. If the inv-ProST layer is left out and the 3D feature maps are directly concatenated instead, the registration accuracy is significantly reduced $(p<10^{-16})$.

## Discussion

In this work, we presented a DL-model for 2D/3D registration, which substantially differs from other DL-methods in the literature. Instead of directly reconstructing the 3D image volume from a pair of DRRs, like in the model of Kasten et al. [7], our network estimates a deformation field that can warp an atlas to the floating space. This has the advantage that large deformations and unlikely shapes can be penalised. Secondly, the estimated deformation field can also be used to warp auxiliary data like label maps. Finally, our network is not restricted to perfect AP and lateral projections.

The comprehensive experiments performed on simulated DRRs from patient CT images show the efficacy of our registration method. The network achieves an average Dice score of 0.94 on the test dataset with orthogonal AP and lateral radiographs. While these biplanar views are the standard in musculoskeletal imaging, the acquisition of perfectly orthogonal AP and lateral radiographs is not always achievable in medical practice. Occasionally, instead of horizontal lateral projections, other lateral views, like the frog-leg or Judet view, are sometimes preferred, depending on the underlying disorder [16]. It was experimentally determined that our method still achieves satisfying results for projection geometries deviating from orthogonality by up to $\pm 10^{\circ }$.

The pair of radiographs that serves as input to our network needs to be calibrated, meaning that the intrinsic and extrinsic parameters of the projection matrix need to be known for both images. The deep network of Gao et al. [9] allow uncalibrated radiographs as input. Their network learns a convex similarity metric with respect to the pose parameters, which is close to the square of geodesic distances in *SE*(3). In the application phase, this convex similarity function can be optimised over the pose parameters by a conventional gradient descent method. It remains a topic of further research to implement this approach to our network in order to enable uncalibrated radiographs as input and to increase the applicability of the network for medical practices.

Furthermore, the input radiographs to our network need to have the femur masked out. While manually annotating the contours would be a subjective and time-consuming task, automatic methods are proposed in the literature to obtain accurate femoral segmentation maps from radiographs [17]. Selection of the region of interest and segmentation would also be an important pre-processing step for registration of more complex anatomical structures.

## Conclusion

This paper presents a novel end-to-end 2D/3D-registration network that registers a 3D atlas image to a pair of radiographs. The network regresses a pose similarity transform and a dense deformation field for local shape variations. It effectively accounts for the projection matrix through a projective and inverse-projective spatial transform layer. The experiments show an average Dice score of 0.94 and an average symmetric surface distance of 0.84 mm on the test dataset, which illustrate the effectiveness of our network.
